# Factor H Binds to Extracellular DNA Traps Released from Human Blood Monocytes in Response to *Candida albicans*

**DOI:** 10.3389/fimmu.2016.00671

**Published:** 2017-01-13

**Authors:** Luke D. Halder, Mahmoud A. Abdelfatah, Emeraldo A. H. Jo, Ilse D. Jacobsen, Martin Westermann, Niklas Beyersdorf, Stefan Lorkowski, Peter F. Zipfel, Christine Skerka

**Affiliations:** ^1^Department of Infection Biology, Leibniz Institute for Natural Product Research and Infection Biology, Jena, Germany; ^2^Research Group Microbial Immunology, Leibniz Institute for Natural Product Research and Infection Biology, Jena, Germany; ^3^Friedrich-Schiller University, Jena, Germany; ^4^Center for Electron Microscopy of the University Hospital Jena, Jena, Germany; ^5^Institute for Virology and Immunobiology, University of Würzburg, Würzburg, Germany; ^6^Institute of Nutrition, Friedrich-Schiller University, Jena, Germany

**Keywords:** *Candida*, monocytes, DNA traps, MPO, factor H

## Abstract

Upon systemic infection with human pathogenic yeast *Candida albicans* (*C. albicans*), human monocytes and polymorph nuclear neutrophilic granulocytes are the first immune cells to respond and come into contact with *C. albicans*. Monocytes exert immediate candidacidal activity and inhibit germination, mediate phagocytosis, and kill fungal cells. Here, we show that human monocytes spontaneously respond to *C. albicans* cells *via* phagocytosis, decondensation of nuclear DNA, and release of this decondensed DNA in the form of extracellular traps (called monocytic extracellular traps: MoETs). Both subtypes of monocytes (CD14^++^CD16^−^/CD14^+^CD16^+^) formed MoETs within the first hours upon contact with *C. albicans*. MoETs were characterized by the presence of citrullinated histone, myeloperoxidase, lactoferrin, and elastase. MoETs were also formed in response to *Staphylococcus aureus* and *Escherichia coli*, indicating a general reaction of monocytes to infectious microbes. MoET induction differs from extracellular trap formation in macrophages as MoETs are not triggered by simvastatin, an inhibitor of cholesterol synthesis and inducer of extracellular traps in macrophages. Extracellular traps from both monocytes and neutrophils activate complement and C3b is deposited. However, factor H (FH) binds *via* C3b to the extracellular DNA, mediates cofactor activity, and inhibits the induction of the inflammatory cytokine interleukin-1 beta in monocytes. Altogether, the results show that human monocytes release extracellular DNA traps in response to *C. albicans* and that these traps finally bind FH *via* C3b to presumably support clearance without further inflammation.

## Introduction

*Candida* spp. are common pathogens in hospital-acquired infections and are the fourth most common cause of nosocomial bloodstream infections (1). Among clinical cases of fungal infections, the most frequently isolated human fungal pathogen is *Candida albicans* (*C. albicans*). This dimorphic human fungus often causes systemic life-threatening infections, particularly in immunocompromised individuals (2, 3). Despite currently available antifungal therapies, *C. albicans-*associated mortality and morbidity remain high (4), and more than 50% of infected patients die due to systemic candidemia (5, 6). Understanding the biology of the infection process and pathogenicity of *C. albicans* will help to find efficient therapies.

Invading fungal cells are immediately attacked by the human innate immune system, which involves the activated complement system, generation of antimicrobial peptides, and stimulation of immune cells. All three complement pathways, the classical, alternative, and lectin pathways, are activated by *C. albicans* (7–9). The relevance of complement activation for the immune response against *C. albicans* is demonstrated by infection of C3- and C5-knockout mice, which show increased mortality as a consequence of impaired anti-*Candida* response and infection-driven immunopathology (10, 11). Induction of the major effector mechanisms in neutrophils was recently demonstrated to be directly dependent on the complement system (12).

In addition, phagocytic and epithelial cells of the innate immune system recognize *C. albicans*. These cells express phylogenetically conserved receptors such as the complement, toll-like, and lectin receptors that bind to pathogen-associated molecular patterns and support phagocytosis and production of pro-inflammatory cytokines and chemokines (13, 14). Among these phagocytic cells, monocytes immediately traffic to sites of *C. albicans* infection, exerting significant candidacidal activity (15). Both classes of monocytes, classical CD14^++^CD16^−^ and non-classical CD14^+^CD16^+^ human blood monocytes, exert candidacidal activity, such as inhibition of germination, phagocytosis, and killing (16). The contribution of monocytes to immune defense was confirmed by *in vivo* studies using mice with depleted mononuclear phagocytes. These mice show accelerated fungal proliferation in tissues and increased mortality (15). The important contribution of monocytes to the innate immune control of infections in blood has been less recognized (17), and candidacidal activity upon first contact remained largely uncharacterized.

Interestingly, monocytes do not substantially contribute to most tissue macrophage populations in the steady state or during certain types of inflammation (18). Instead, they are considered to represent precursors of peripheral mononuclear phagocytes with the central functions of uptake of microbes and induction of cytokines. However, monocytes are besides neutrophils the first cells that come into contact with microbes in the blood and may have additional functions in innate immunity. In this study, we show how monocytes exert their early anti-fungicidal activities, which include phagocytosis of the microbe, generation of myeloperoxidase (MPO) to attack the pathogen, and formation of extracellular DNA traps to restrict dissemination of the fungus. Extracellular DNA in form of monocytic extracellular traps (MoETs) from monocytes or neutrophil extracellular traps (NETs) from neutrophils activates complement and C3b deposits onto MoETs and NETs. Interestingly, complement factor H (FH) binds *via* C3b to the extracellular DNA, retains cofactor activity for factor I, and restricts complement activation as well as further inflammation.

## Materials and Methods

### Cells

Monocytes were isolated from fresh sterile buffy coats (Jena University Hospital, Germany) or from fresh blood collected from healthy volunteers after informed consent according to guidelines from the local ethics committee. The study was conducted in accordance with the Declaration of Helsinki, all the protocols were approved by the Ethics Committee of the Jena University Hospital. Human PBMCs were isolated by Ficoll-Paque PLUS (GE healthcare, Freiburg, Germany) (density 1.077 g/ml) density gradient centrifugation. Lymphocytes were removed from the isolated PBMCs using 46% Percoll (GE healthcare, Freiburg, Germany) density gradient centrifugation. Monocytes were further purified according the manufacturer’s protocol provided with pan monocyte isolation kit (Miltenyi Biotec, Teterow, Germany). Isolated monocytes were suspended in growth medium (RPMI medium supplemented with 10% heat-inactivated FCS and 2 mM ultra glutamine 1) and used for experiments within 1 h. Purity of isolated cells was confirmed by identifying CD14 (FITC anti-human CD14 antibody, Biolegend, London, UK) on the cells using flow cytometry.

Monocytic cells THP-1 (ATCC 16, Manassas, VA, USA) were maintained in growth medium at 37°C and 5% CO_2_. Cells were passaged every 2 days and used for experiments until 30th passage. Complement active human serum was prepared from fresh whole blood, which was immediately centrifuged (10 min, 13,200 rpm, 4°C) mixed in a pooled stock and stored in aliquots at −80°C.

### Microbial Strains and Culture

*Candida albicans* wild type (SC5314) (19) and GFP-expressing *C. albicans* (20) were grown overnight in yeast extract–peptone–dextrose medium (2% d-glucose, 1% peptone, 5% yeast extract in water) at 30°C, reseeded in yeast–peptone–dextrose medium, grown for 4 h at 30°C into the midlog-phase. *Staphylococcus aureus* (USA 300 LAC) and *Escherichia coli* (XL1-Blue) were grown overnight in tryptic soy broth and Luria–Bertani broth at 37°C, reseeded in same broth and grown until OD 1 at 37°C into the midlog-phase.

### Phagocytosis Assay

GFP-expressing *C. albicans* (5 × 10^6^) were added to monocytes (5 × 10^5^) in a 24-well plate in growth medium or in growth medium with complement active normal human serum (NHS, 10%). Monocytes were pre-stained with Vybrant DiD Cell-Labeling solution (5 µM) (Thermo Fisher Scientific, Dreieich, Germany). After 0–2 h of coincubation at 37°C with 5% CO_2_, *C. albicans* were collected by removing the supernatant and monocytes were collected by detachment of cells using accutase (GE healthcare, Freiburg, Germany). Phagocytosis of *C. albicans* by monocytes was analyzed by flow cytometry. In parallel, *C. albicans* cells were labeled with mouse monoclonal Pra1 antibodies, and phagocytosis was measured as described above. Monoclonal antibodies (mAb) were generated by standard methods and specificity of the mAb was confirmed by Western blotting (data not shown).

### Microscopy

#### Live Cell Imaging

Monocytes (2 × 10^5^) were seeded on Poly-l-lysine coated 30 mm culture dishes and incubated in growth medium with 10% NHS at 37°C with 5% CO_2_. *C. albicans* wild type (SC5314) or GFP-expressing *C. albicans* (each 2 × 10^5^) were added to the seeded cells. For detection of phagocytosis, monocytes were pre-stained with Vybrant DiD Cell-Labeling solution (5 µM) and DNA release was detected with nucleic acid dye SYTOX blue (Thermo Fisher Scientific, Dreieich, Germany) (5 µM) during coincubation. Monocyte subgroups were detected with Alexa Fluor 488 labeled anti-human CD14 antibody (1:100) and Alexa Fluor 647 labeled anti-human CD16 antibody (each 1:100) (both Biolegend, London, UK). Monocytes were co-incubated in small culture dishes at 37°C with 5% CO_2_ and subjected to confocal laser scanning microscopy (LSM 710 from Carl Zeiss, Jena, Germany). Live time images were captured over 6 h taking images every 30 s using ZEN 2011 (magnification 40×, 444/480 nm for SYTOX blue, 488/509 nm for GFP, 488/525 nm for Alexa Fluor 488, 594/633 nm for Alexa Fluor 647, and 650/670 nm for DiD dye). The experiments were repeated two to seven times.

#### Fixed Cell Imaging

*Candida albicans* wild type (SC5314) or Calcofluor-white stained (Sigma-Aldrich, Taufkirchen, Germany) *C. albicans* wild type (SC5314) were added to monocytes (5 × 10^5^) on 13 mm Poly-l-lysine coated coverslips in a 24-well plate in growth medium with 10% NHS. Monocytes were also incubated with 50 µg/ml whole glucan particles (WGP) (Invivogen, San Diego, CA, USA) or 20 ng/ml LPS (Sigma-Aldrich, Taufkirchen, Germany). Upon co-incubating monocytes with *C. albicans* or WGP or LPS for 4 h at 37°C in 5% CO_2_, media were aspirated, and reaction was fixed using 4% paraformaldehyde (PFA) for 10 min. For degradation of MoETs, probes were treated with 10 U/ml of DNAse-1 (Thermo Fisher Scientific, Dreieich, Germany) for 30 min before adding 4% PFA. After treatment with PFA, coverslips were stained with 5 µM SYTOX green for the detection of MoETs or coverslips were blocked for 1 h in 10% FCS, 1% BSA, and 0.1% Tween 20 in PBS. Cit-H3 on MoETs was detected by incubating the probes for 1 h with rabbit anti-histone H3 (citrulline R2 + R8 + R17) antibody (Abcam, Cambridge, UK) (1:1,000) followed by Alexa Fluor 647 conjugated anti-rabbit IgG (H + L) secondary antibody (Thermo Fisher Scientific, Dreieich, Germany) (1:2,000) and SYTOX green (5 µM). MPO was detected with mouse anti-human MPO antibody (Biolegend, London, UK) (1:500), elastase with mouse anti-human elastase (AbD Serotec, Puchheim, Germany) (1:500), and lactoferrin (LAC) with mouse anti-human LAC (AbD Serotec, Puchheim, Germany) (1:500) antibodies all followed by staining with Alexa Fluor 647 labeled anti-mouse IgG (H + L) secondary antibody (Thermo Fisher Scientific, Dreieich, Germany) (1:2,000) and SYTOX green (5 µM). CD14 on MoETs was detected by incubating the probes for 1 h with Alexa Fluor 488 anti-human CD14 Antibody (1:100) and SYTOX orange (5 µM) (Thermo Fisher Scientific, Dreieich, Germany). C3b on MoETs and NETs was detected using rabbit anti-human complement C3d antibody (Dako, Hamburg, Germany) (1:100) and C5b–9 on MoETs was detected using rabbit anti-human Complement SC5b–9 neoantigen antiserum (Comptech, TX, USA) (1:100) then followed by staining with Alexa Fluor 647 conjugated anti-rabbit IgG (H + L) secondary antibody (Thermo Fisher Scientific, Dreieich, Germany) (1:2,000) and SYTOX green (5 µM). FH on MoETs and NETs was detected using goat anti-human FH anti serum (Comptech, TX, USA) (1:100) followed by staining with Alexa Fluor 647 conjugated with anti-goat IgG (H + L) secondary antibody (Thermo Fisher Scientific, Dreieich, Germany) (1:2,000) and SYTOX green (5 µM). Images were captured using LSM 710 equipped with ZEN 2011 (355/433 nm for Calcofluor-white, 488/525 nm for Alexa Fluor 488, 504/523 nm for SYTOX green, 547/570 nm for SYTOX orange, and 594/633 nm for Alexa Fluor 647). MoETs from monocytes were similarly detected in presence of *S. aureus* (USA 300 LAC) and *E. coli* (XL1-Blue) with a MOI of 1:10. The experiments were repeated three to five times.

#### Electron Microscopy

Monocytes (5 × 10^5^) alone or monocytes (5 × 10^5^) with *C. albicans* wild type (SC5314) (5 × 10^5^) were added to a 12 mm Poly-l-lysine coated coverslips placed in a 24-well plate in growth medium with 10% NHS. After 4 h of coincubation at 37°C with 5% CO_2_, media were aspirated, and reaction was pre-fixed for 1 h using 2.5% glutaraldehyde in sodium cacodylate buffer (0.1 M, pH 7.0). The samples were washed with cacodylate buffer and post-fixed for 1 h with 1% osmium tetroxyde in cacodylate buffer. Samples were dehydrated in rising ethanol concentrations followed by critical point drying in a Leica EM CPD300 Automated Critical Point Dryer (Leica, Wetzlar, Germany) and finally coated with platinum (6 nm) in a BAL-TEC MED 020 Sputter Coating System (BAL-TEC, Balzers, Liechtenstein). The samples were imaged at different magnifications with a Zeiss-LEO 1530 Gemini field emission scanning electron microscope (Carl Zeiss, Oberkochen, Germany) at 7 kV acceleration voltage and a working distance of 7 mm using an intense secondary electron detector. The experiment was repeated three times.

### DNA Quantification Assay

Previously described procedure was followed for this quantification (21). Shortly, monocytes (10^5^ cells/well) were seeded into 96-well black plates. For coincubation, *C. albicans* (5 × 10^5^ cells/well) was added to each well at a MOI of 1:5 in growth medium alone or in growth medium with 10% NHS or with 10% heat-inactivated NHS (56°C for 1 h). No *C. albicans* was seeded for control monocytes. SYTOX green (5 µM) was added to all probes and incubated at 37°C with 5% CO_2_. DNA release and ROS formation was quantified by measuring fluorescence intensity of SYTOX green (485/538 nm) every hour using a Safire plate reader (Tecan, Männedorf, Switzerland) and subtracting the values of fluorescence intensity of control monocyte. Similarly, DNA was quantified from neutrophils. The experiments were repeated three to four times.

### Isolation of Extracellular Traps

Extracellular traps from monocytes were isolated according to a modified protocol by Barrientos et al. (22). Briefly, monocytes (1.5 × 10^6^) were seeded into 12-well plates and incubated with *C. albicans* cells (7.5 × 10^6^) in growth medium with 10% NHS for 4 h at 37°C and 5% CO_2_. Monocytes alone were used as controls. Following 4 h incubation period, the wells were washed twice by DPBS, and probes were incubated with *Alu*I (4 U/ml) (Thermo Fisher Scientific, Dreieich, Germany) for 20 min at 37°C to digest the extracellular traps into fragments. The supernatants were harvested by centrifugation (10,000 rpm for 5 min at 4°C) and the amount of extracellular DNA was quantified after staining with SYTOX green (5 µM). For standardization, Lambda DNA (Thermo Fisher Scientific, Dreieich, Germany) was similarly stained and measured (485/538 nm). Neutrophils were incubated with *C. albicans* or 100 nM phorbol 12-myristate 13-acetate (PMA) (Sigma-Aldrich, Taufkirchen, Germany) in growth medium and extracellular traps were isolated. Presence of citrullinated H3 was also assessed by Western blot analysis of isolated MoETs using rabbit anti-histone H3 (citrulline R2 + R8 + R17) antibody (Abcam, Cambridge, UK) (1:1,000) and polyclonal goat anti-rabbit antibody/HRP (Dako, Hamburg, Germany) (1:2,000).

### Antifungal Assay

Freshly cultured wild-type *C. albicans* cells (5 × 10^3^) were incubated with 50 ng of isolated MoET DNA or 50 ng of THP-1 chromosomal DNA or 50 ng of monocyte chromosomal DNA in growth medium at 37°C for 30 h inside Spectra Max 190 plate reader. Chromosomal DNA was isolated using PAXgene blood DNA kit (Qiagen, Venlo, Netherlands). *C. albicans* growth was measured every 10 min for 30 h by measuring absorbance at 604 nm using the SOFTmax PRO software. The experiment was repeated five times.

### ELISA

Immobilization of NETs on 96-well plates and blocking was performed according to a modified protocol by Caudrillier et al. (23). Briefly, rabbit anti-human MPO antibodies (Sigma-Aldrich, Taufkirchen, Germany) (1:100) or rabbit anti-human histone H3 (citrulline R2 + R8 + R17) antibodies (Abcam, Cambridge, UK) (1:200) were coated onto 96-well plates overnight at 4°C. Blocking was performed with 5% BSA and 0.1% human serum albumin in DPBS. Isolated NETs were added and incubated at room temperature for 1 h. NHS- or C3-depleted sera or alternative pathway activated NHS (by addition of 10 mM EGTA) or complement inhibited NHS (by the addition of 10 mM EDTA) or purified FH (5–20 μg/ml) and/or C3b (20 µg/ml) (both Comptech., TX, USA) were added and incubated for 1 h at 37°C. FH and C3b were detected on the extracellular traps using goat anti-human FH or goat anti-human C3 antiserum (Comptech, TX, USA) (1:5,000) followed by rabbit anti-goat antibody/HRP (Dako, Hamburg, Germany) (1:5,000). The experiments were repeated three to five times.

Interleukin-1 beta (IL-1β) generation by monocytes was measured with IL-1β ready-set-go ELISA kit (eBioscience, Frankfurt, Germany) according to the manufacturer’s protocol. Blood-derived monocytes were incubated with similar amounts of isolated NETs in growth medium (also with 10% NHS) for 20 h and IL-1β was determined in the supernatants by the ELISA kit. The amount of IL-1β produced by monocytes in presence of NETs with FH (12.5 µg/ml) or together with C3b (12.5 µg/ml) was also determined. The experiment was repeated three times.

### Complement Cofactor Assay

Neutrophil extracellular traps were immobilized on a microtiter plate as described before (23) and incubated with C3b (20 µg/ml) and FH (5–20 µg/ml) or NHS (10%) for 30 min at RT. After washing off unbound FH, C3b (10 µg/ml) and factor I (7 µg/ml) (Comptech, TX, USA) were added and incubated at 37°C for 1 h. The reaction was stopped with the addition of Roti-Load 1 (Carl Roth, Karlsruhe, Germany), and proteins were separated by SDS-PAGE. C3b cleavage was assessed by Western blot analysis using goat complement C3 antiserum (Comptech, TX, USA) (1:1,000) followed by rabbit anti-goat antibody/HRP (1:2,500). The experiment was repeated three times.

### Mouse Model of Disseminated Candidiasis

All animal experiments were conducted in compliance with European and German regulations. Protocols were approved by the responsible Federal State authority and ethics committee (Thüringer Landesamt für Verbraucherschutz, permit number: 03-006/09). Female BALB/c mice (Charles River, Germany) weighing 18–20 g were housed in groups of five in individually ventilated cages with free access to water and food. For infection, *C. albicans* was grown for 12 h at 30°C in YPD medium, washed three times in sterile PBS, and diluted to the desired concentrations. The infection dose was confirmed by plating. On day 0, the mice were infected *via* the lateral tail vein with 2.5 × 10^4^ *C. albicans* cfu/g body weight. The health status of the mice was examined at least twice daily. Liver was collected 6 and 24 h post-infection, fixed in 10% neutral buffered formalin (Histofix, Carl Roth, Karlsruhe, Germany), embedded in paraffin, and sectioned at 4 µm thickness.

### Immunohistochemistry of Liver Sections Derived from Mice with Disseminated Candidiasis

Paraffin-embedded mice liver sections were deparrafinized by placing them consecutively in Roticlear (Carl Roth, Karlsruhe, Germany), 100% ethanol, and 95% ethanol. Sectioned tissues were boiled in 10 mM Na citrate buffer (pH 6.5) for antigen retrieval and blocked in 1% BSA supplemented PBS for 30 min. For the detection of CD115, Ly-6C, and Ly-6G in tissues from 6 h post-infected mice, samples were treated with Alexa Fluor 488 anti-mouse CD115 (CSF-1R) Antibody (1:100), Alexa Fluor 700 anti-mouse Ly-6C Antibody (1:100), and Brilliant Violet 421 anti-mouse Ly-6G Antibody (1:100), respectively (all three antibodies are from Biolegend, London, UK). About 0.1% saponin supplemented PBS was used to dilute antibodies, and SYTOX orange was used to detect DNA in the tissue samples. Alexa Fluor 350 labeled anti-mouse FH antibody (1:1,000) (Bioss antibodies, MA, USA) diluted in 0.1% saponin supplemented PBS was used for detection of FH deposition in 24 h post-infected tissues and SYTOX green (5 µM) was used to detect DNA in the probes. Images were captured using LSM 710 with ZEN 2011 (401/421 nm for Alexa Fluor 350 and Brilliant Violet 421, 488/525 nm for Alexa Fluor 488, 504/523 nm for SYTOX green, 547/570 nm for SYTOX orange, and 679/702 nm for Alexa Fluor 700). The experiment was repeated three times.

### Statistical Analysis

Significant differences between two groups were analyzed using the Student’s two-tailed *t*-test or one way ANOVA. Values of *p < 0.05, **p < 0.01, and ***p < 0.001 were considered statistically significant.

## Results

### Monocytes Phagocytose *C. albicans* Cells and Release Extracellular DNA

**Figure 1:**
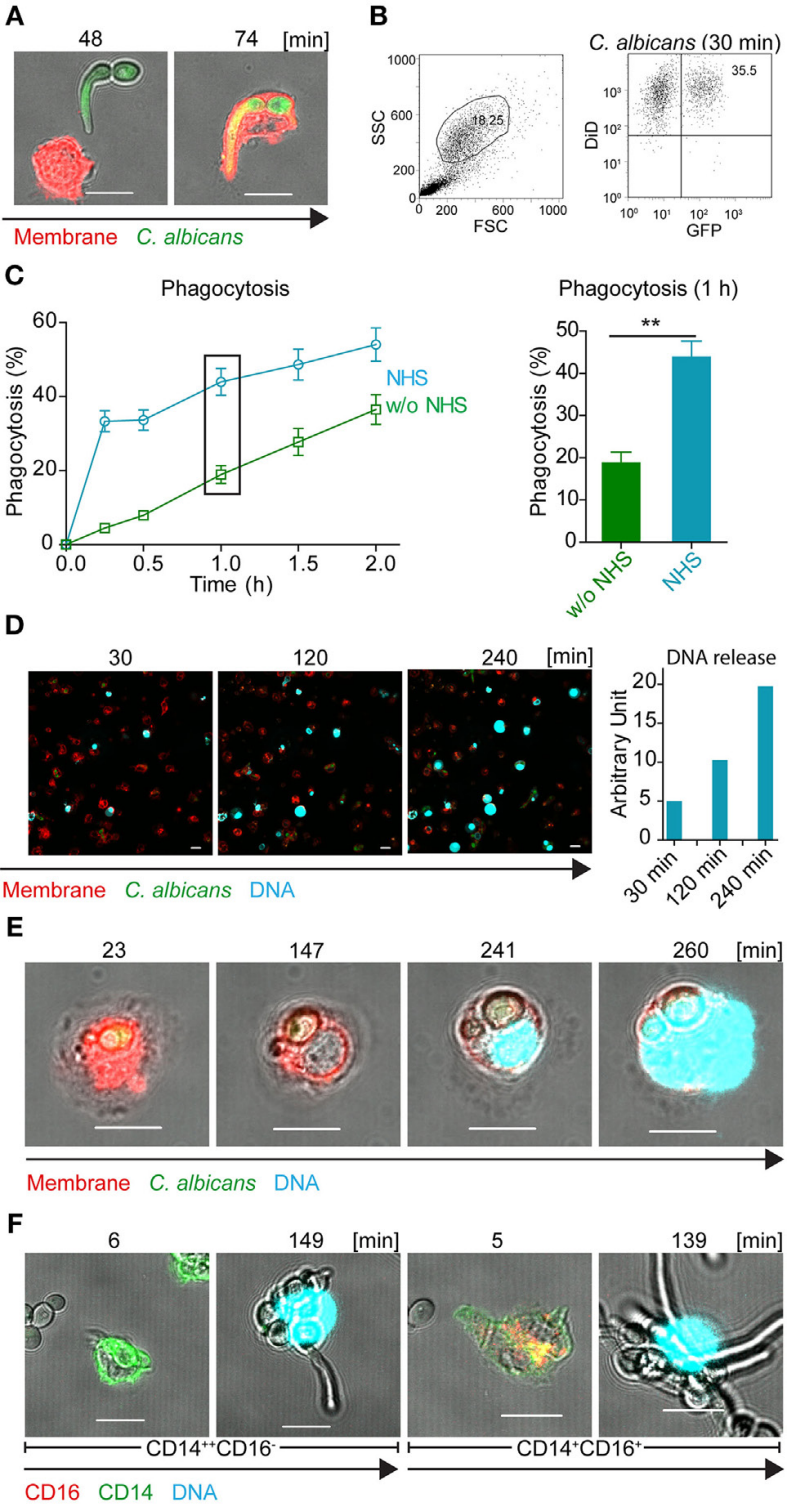
**Human blood-derived monocytes release DNA in response to *Candida albicans***. **(A)** Human blood monocytes (red) spontaneously engulf GFP-*C. albicans* cells (green). Representative cells from three independent experiments are shown. Pictures were taken with a LSM710 microscope (Zeiss), fitted with a 40×, 1.4 NA, oil-immersion lens, and processed, using ZEN 2011 software (Zeiss). Scale bar: 10 µm. **(B)** Representative example of gating strategy to determine by flow cytometry single- and double-positive monocytes (GFP and DID). **(C)** Phagocytosis of GFP-*C. albicans* is enhanced in complement active human serum (NHS). GFP-expressing *C. albicans* were incubated with monocytes in the absence or presence of human serum and the phagocytosis rate was determined by flow cytometry. Data represent mean values ± SDs of four independent experiments (Phagocytosis in NHS versus iNHS after 1 h **p = 0.0083; one sided Student’s *t*-test). **(D)** Peripheral blood monocytes react on GFP-*C. albicans* by the release of DNA, which was stained with SYTOX blue (upper panel). Release of DNA increased over time (lower panel) as measured by ZEN 2011 software (Zeiss). **(E)** Human blood monocytes phagocytose *C. albicans* cells, decondense after about 150 min the chromosomal DNA, and subsequently burst out the DNA. Blood monocytes were incubated with GFP-*C. albicans* cells at a ratio of 1:1 in the presence of 10% complement active human serum and interaction was followed by life time imaging using LSM (see also Video S1 in Supplementary Material). Red: DiD stained membrane, blue: SYTOX blue, green: GFP-*Candida*. **(F)** Both types of monocytes CD14^++^CD16^−^ and CD14^+^CD16^+^ release DNA in response to *C. albicans* at about 150 min. Green: CD14, red: CD16, blue: SYTOX blue. Representative cells from at least three independent experiments. Pictures were taken with an LSM710 microscope (Zeiss), fitted with a 40×, 1.4 NA, oil-immersion lens, and processed, using ZEN 2011 software (Zeiss). Size bars: 10 µm.

To follow the direct response of human monocytes to *C. albicans in vitro*, live cell imaging was performed using laser scanning microscopy. Human monocytes were isolated from human blood using MACS (purity about 95% according to CD14 expression). These monocytes were incubated with GFP-expressing *C. albicans* at a ratio of 1:1 in growth medium with NHS in the presence of SYTOX blue, a nucleic acid dye that does not cross the intact cell wall membrane. Interaction of monocytes with *C. albicans* was followed over 12 h. Monocytes migrated toward the fungal pathogen and engulfed *C. albicans* cells within minutes (Figure 1A). Phagocytosis in 10% NHS, as evaluated by flow cytometry, was enhanced (about 30% after 15 min) compared with that in growth medium alone (Figures 1B,C).

Within the first minutes of contact with *C. albicans*, monocytes were highly active and engulfed *C. albicans* cells. During the next 2–4 h of cultivation, several monocytes showed morphological changes. The nuclei enlarged, and faint staining of nuclear DNA appeared. This staining is explained by increased membrane permeability and influx of the DNA dye SYTOX blue (Figures 1D,E). During the following 2 h, nuclear DNA decondensed and in several cases the DNA was suddenly released from the monocyte and appeared in the extracellular compartment around the ruptured membrane and on *C. albicans* (Video S1 in Supplementary Material; Figures 1D,E). In single cases, membrane rupture and release of extracellular DNA was detected even earlier, after 20–40 min. As cell remnants appeared in the medium, this type of DNA release resulted in monocyte cell death.

To compare this DNA release by monocytes with the previously described extracellular trap formation by neutrophils (24), the response of neutrophils to *C. albicans* was followed under identical experimental conditions. Neutrophils that were challenged with live *C. albicans* cells migrated toward the fungus, engulfed the pathogen, and also released the DNA in about the same time frame as monocytes (Figure S1 in Supplementary Material). Thus, both human innate immune cells, monocytes and neutrophils, release DNA within the first hours upon contact with *C. albicans*.

To determine whether release of DNA in response to *C. albicans* is specific to one subtype of monocytes or whether both types of monocytes (CD14^++^CD16^−^/CD14^+^CD16^+^) burst and release nuclear DNA, blood monocytes were again incubated with *C. albicans* in the presence of complement active human serum together with the DNA marker SYTOX blue. Monocytes were stained for CD14 and CD16 to allow identification of the specific subtype. Upon contact with *C. albicans*, both types of monocytes (CD14^++^CD16^−^ and CD14^+^CD16^+^) released DNA within 2–3 h (Figure 1F).

### DNA Released by Monocytes Captures *C. albicans*

**Figure 2:**
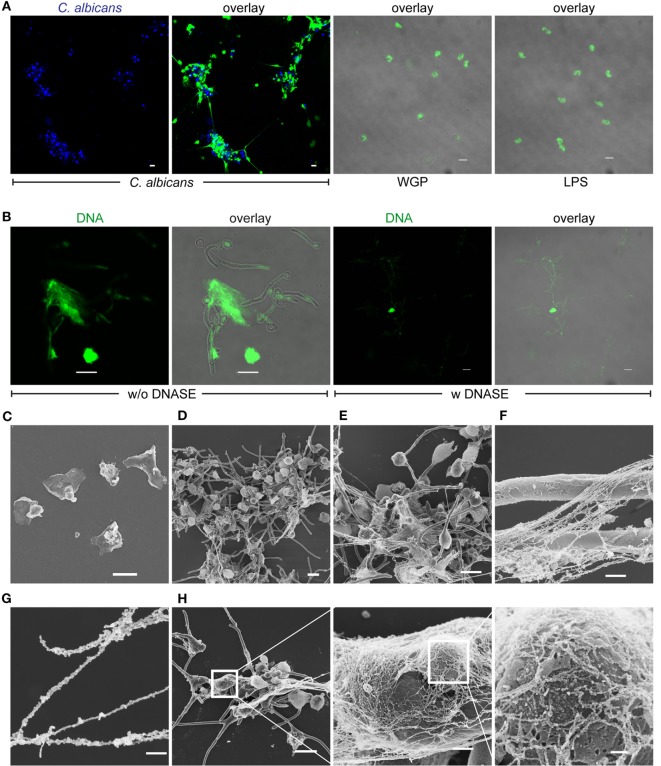
**Released DNA in response to human monocytes traps *Candida albicans***. **(A)** DNA released by monocytes forms traps and immobilizes *C. albicans*, whereas whole glucan particles (WGP) or LPS have no effect. Blue: *C. albicans*, green: SYTOX green. **(B)** DNAse treatment leads to digestion and disappearance of DNA traps. Green: SYTOX green. Pictures in **(A,B)** were taken with an LSM710 microscope (Zeiss), fitted with 20× 1.4 NA lens or 40× 1.4 NA oil-immersion lens or 63× 1.4 NA oil-immersion lens, and processed, using ZEN 2011 software (Zeiss). Scale bar: 10 µm. Scanning electron microscopy of **(C)** blood monocytes alone after 4 h. Scale bar: 10 µm. **(D,E)** DNA fibers and nets that trap *C. albicans* cells and also hyphae. Scale bar: 10 µm. **(F)** Monocytic extracellular traps (MoETs) form dense structures that cover hyphae. Scale bar: 1 µm. **(G)** MoETs are covered with proteins and form strings with bead-like structures. Scale bar: 200 nm. **(H)** Magnification of MoETs demonstrates a dense network that covers *C. albicans* and damages the surface. Scale bars = 10 μm, 1 µm, and 200 nm.

Having shown that, upon contact with *C. albicans*, monocytes respond to the fungal pathogen by undergoing nuclear changes and releasing their DNA, the extracellular DNA was further characterized. Monocytes were cultivated with *C. albicans* in growth medium with 10% NHS for 4 h then fixed and stained for DNA with SYTOX and evaluated by microscopy. The released extracellular DNA appeared as fibrous structures, which were formed between several monocytes and *C. albicans* (Figure 2A). The extracellular DNA release was triggered by *C. albicans* but not after addition of WGP or LPS. To exclude DNA release of monocytes by piercing of *C. albicans* formed hyphae monocytes were also incubated with hyphae locked mutant *C. albicans* strain (cph1Δ/efg1Δ). Monocytes also formed extracellular DNA traps within 2–4 h coincubation with mutant *C. albicans* cells (Figure S2 in Supplementary Material). Upon DNase treatment, the extracellular traps disappeared, confirming DNA comprising the traps (Figure 2B). The shape and appearance of this extracellular DNA was further determined by electron microscopy. The extracellular DNA released by monocytes (Figure 2C) formed cluster-like structures where groups of *C. albicans* were intertwined (Figure 2D). Furthermore, extracellular DNA formed dense nets, which were comprehensively deposited onto the surface of *C. albicans* cells and hyphae (Figures 2E,F). In addition, bead-like structures appeared on the DNA strings (Figure 2G), which most likely represent multiple proteins. These extracellular DNA covered the fungal surfaces (Figure 2H). These results demonstrate that monocytes liberate DNA to form extracellular trap-like structures, which we call MoETs that immobilize *C. albicans* cells and hyphae.

Extracellular trap formation is originally described for neutrophils and represents a specific response of suicidal cell death, which is distinct from apoptosis (25). To rule out the possibility that monocytes undergo rapid apoptosis and secondary necrosis in response to *C. albicans*, cellular morphology was also studied in monocytes undergoing apoptosis. Upon induction of apoptosis, blebs appeared at around 2.5 h on the cell surface. In contrast to trap forming monocytes nuclear DNA condensed followed by secondary necrosis and extracellular DNA after 5 h (Figure S3 in Supplementary Material). In summary, DNA release by monocytes *via* so-called MoETosis is a specific reaction by monocytes to combat microbes and is morphologically different to apoptosis.

### DNA Released by Monocytes Contains MPO, Elastase (E), Citrullinated Histone H3 (cit H3), LAC, and Monocyte Marker CD14

**Figure 3:**
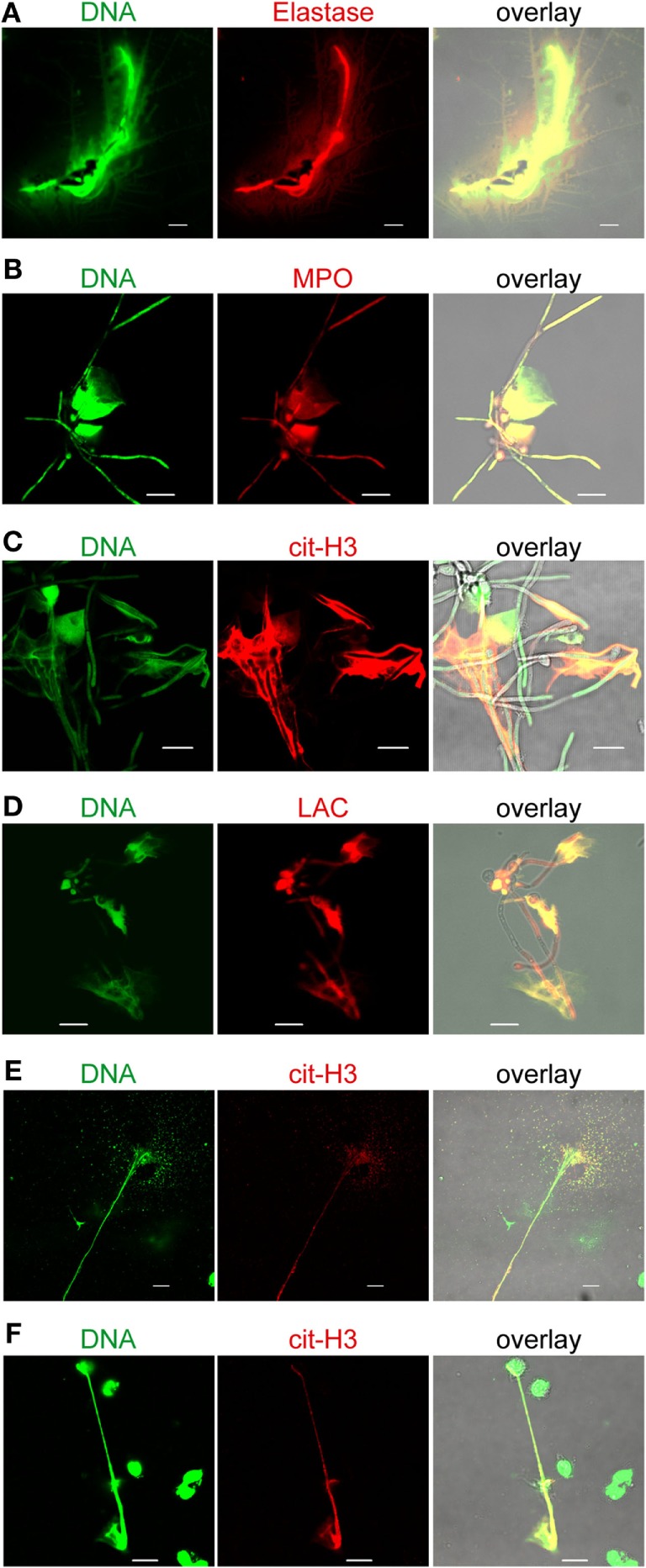
**Monocytic extracellular traps stain positive for citrullinated histone (H3) myeloperoxidase (MPO), elastase (E), and lactoferrin (LAC)**. Extracellular traps derived from monocytes contain **(A)** elastase, **(B)** MPO, **(C)** citrullinated H3 (cit-H3), and **(D)** LAC, which are known to harbor antimicrocidal activities. Human blood monocytes were incubated with *Candida albicans* (1:1) in 10% normal human serum for 4 h, fixed, and stained with specific antibodies for LS microscopy. Green: SYTOX green, red: citrullinated H3, or MPO, or elastase or lactoferrin as marked. **(E)** Monocytes incubated with Gram-positive (*Staphylococcus aureus*) or **(F)** Gram-negative (*Escherichia coli*) bacteria also form extracellular traps (green). Additionally, these traps contain citrullinated H3 (cit-H3, red). Representative cells from at least three independent experiments. Pictures were taken with an LSM710 microscope (Zeiss), fitted with a 63×, 1.4 NA, oil-immersion lens, and processed, using ZEN 2011 software (Zeiss). Scale bar: 10 µm.

In neutrophils, ROS trigger the dissociation of neutrophil elastase (NE) from a membrane-associated complex into the cytosol and activate its proteolytic activity in a MPO-dependent manner (26). NE is stored in azurophilic granules and contributes to antimicrobial activity in the phagosome. During NET formation, NE translocates from the granules to the nucleus and cleaves histones to promote chromatin decondensation. In this process, histone H3 becomes citrullinated and is liberated together with the DNA. To investigate whether MPO and elastase are active in MoETosis and whether histones are citrullinated in MoETs, extracellular DNA of monocytes was stained for serine protease elastase, MPO, and cit H3. MoETs were positively stained for all three proteins (Figures 3A–C). Similarly, MoETs were positively stained for LAC, a granule protein with antimicrobial activity (27) (Figure 3D). MPO, elastase, cit H3, and LAC within the extracellular traps covered the surface of *C. albicans* cells and hyphae. These results demonstrate that the DNA released from monocytes in response to *C. albicans* represents nuclear DNA and comprises antimicrobial proteins. Thus, the mechanism of MoETosis described here for monocytes is related to NETosis in neutrophils.

To clarify whether DNA release by monocytes is a specific reaction to *C. albicans* or a more general response to infectious microbes, monocytes were incubated with either the Gram-positive bacterium *S. aureus* or the Gram-negative bacterium *E. coli*. Using the same experimental setup as employed with *C. albicans*, monocytes were fixed after 4 h of incubation and DNA release was determined by laser scanning microscopy. Monocytes engulfed both the Gram-positive and Gram-negative bacteria and also released nuclear DNA (Figures 3E,F). Thus, human blood monocytes respond to the microbes *C. albicans, S. aureus*, and *E. coli* by spontaneous DNA release. Furthermore, the liberated DNA contained cit H3, a specific marker for extracellular traps.

**Figure 4:**
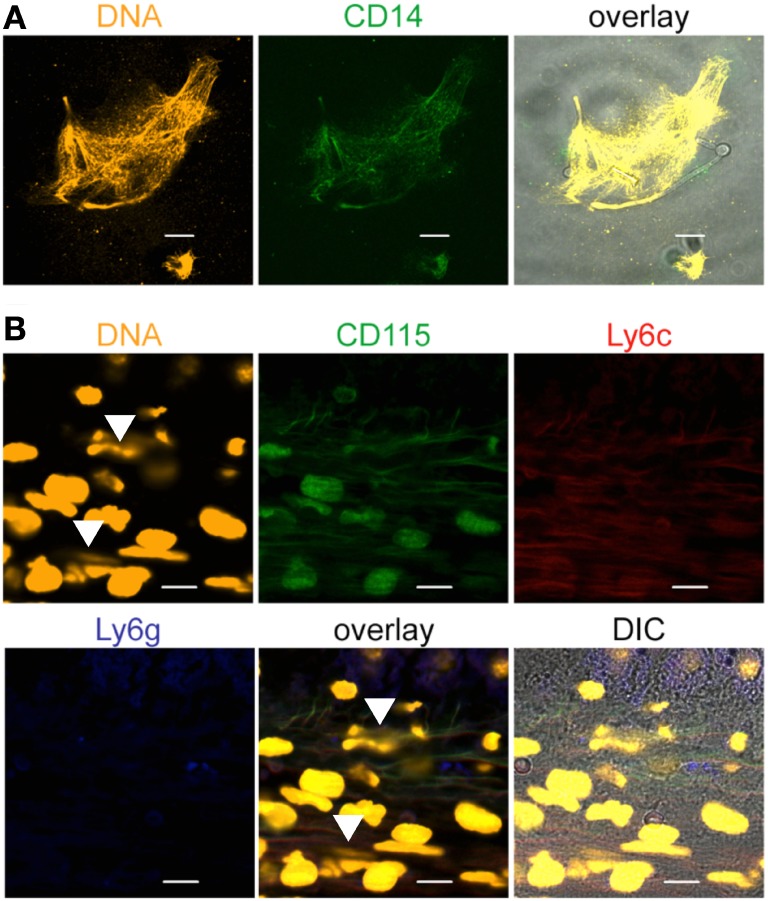
**Monocytic extracellular traps stain positive for CD14, CD115, and Ly6c**. Extracellular traps derived from monocytes contain the monocytic marker **(A)** CD14. Human blood monocytes were incubated with *Candida albicans* (1:1) in growth medium with 10% NHS for 4 h, fixed, and stained with specific antibodies. Orange: SYTOX orange, green: CD14. **(B)** Mouse monocytes form extracellular traps (arrows) in mouse liver sections derived from *C. albicans*-infected mice. The traps show the monocytic markers CD115 and Ly6c, but not Ly6g, a marker for neutrophils. Representative stainings from at least three independent experiments are shown. Orange: DNA (SYTOX orange), green: CD115, red: Ly6c, blue: Ly6g. Pictures were taken with an LSM710 microscope (Zeiss), with 40× 1.4 NA oil-immersion lens or 100× 1.4 NA oil-immersion lens, and processed, using the ZEN 2011 software (Zeiss). Scale bar: 10 µm.

To confirm monocyte origin of released DNA in response to *C. albicans*, monocytes and released DNA was stained for presence of CD14, a surface marker which is abundant on human monocytes and much less expressed on human neutrophils (Figure S4 in Supplementary Material). The monocyte and the released DNA showed strong CD14 staining after 4 h of coincubation with *C. albicans* (Figure 4A), confirming the monocytic origin. In order to confirm DNA release by monocytes *in vivo* formalin-fixed liver tissues from 6 h *C. albicans*-infected mice were stained with the monocytic markers CD115 and Ly6c, the granulocyte marker Ly6g and DNA stain SYTOX orange. Extracellular DNA was identified in the liver tissue which stained for CD115 and Ly6C (both markers for monocytes) and absence of Ly6G (marker for neutrophils) (Figure 4B). These data confirm *in vivo* trap formation.

### Monocytic Extracellular DNA Inhibits Growth of *C. albicans*

**Figure 5:**
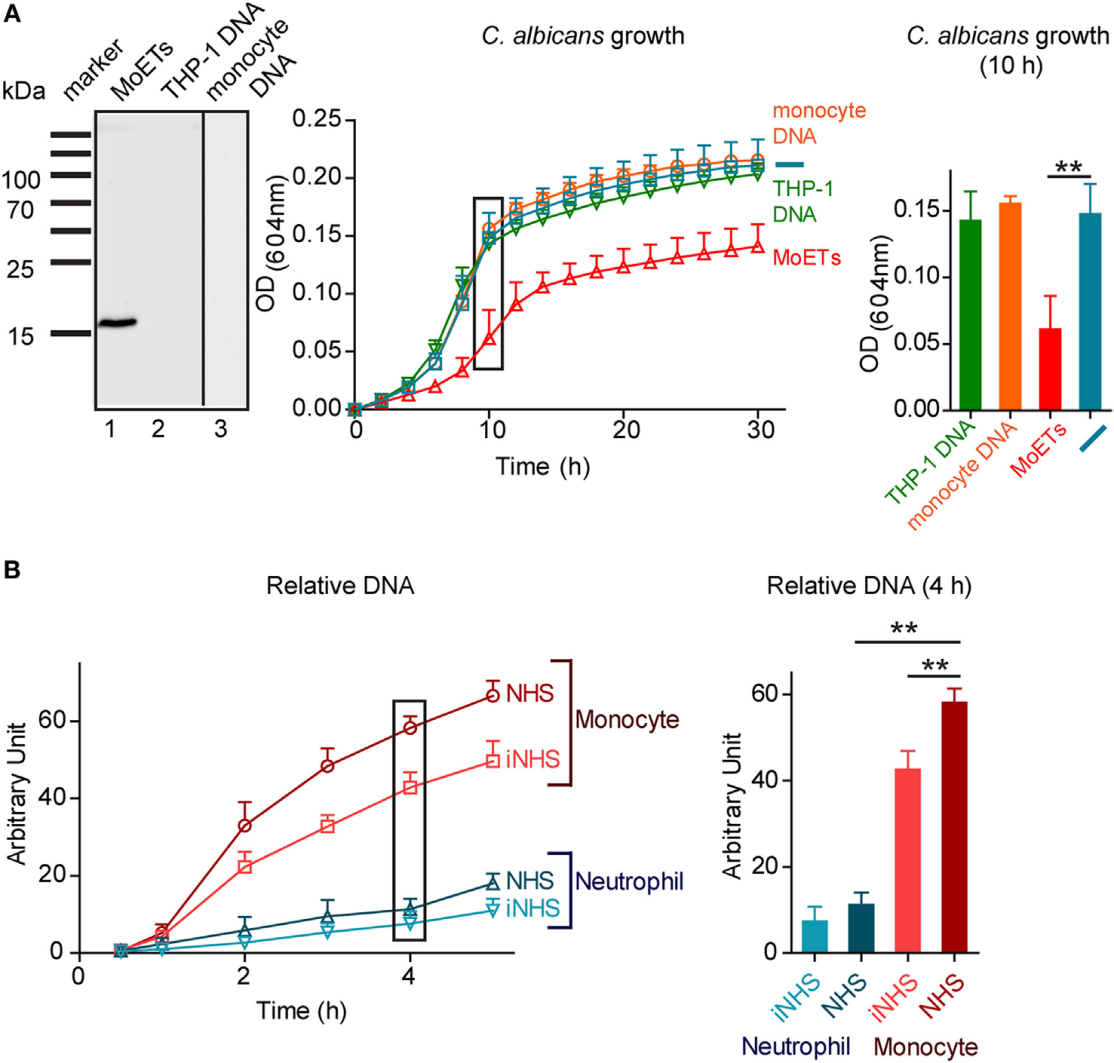
**Extracellular DNA released by monocytes inhibits growth of *Candida albicans***. **(A)** Isolated monocytic extracellular traps (MoETs) stain for citrullinated H3 in Western blot analysis (left panel). Isolated MoETs kill *C. albicans* by about 45% after 12 h of coincubation (middle and right panel). In contrast, addition of chromosomal DNA derived from blood monocytes or THP-1 cells had no inhibitory effect on the growth rate of *Candida*. Data represent mean values ± SDs of five independent experiments (inhibition with MoETs versus no or chromosomal DNA, **p = 0.0067; two sided Student’s *t*-test). **(B)** Human monocytes release DNA in response to *C. albicans*, which is enhanced in complement active human serum. In comparison human neutrophils release less DNA in response to *C. albicans*. Data represent mean values ± SDs of four independent experiments [monocytes in normal human serum (NHS) versus iNHS **p = 0.0056, monocytes versus neutrophils in NHS **p = 0.0020; two sided Student’s *t*-test].

Having shown that monocytes release DNA upon contact with the pathogenic fungus *C. albicans*, we asked whether this extracellular DNA affects pathogen survival. To this end, extracellular DNA released by monocytes upon incubation for 4 h with *C. albicans* was isolated. The isolated DNA and the associated proteins were separated by SDS-PAGE, proteins were immunoblotted, and the presence of cit H3 was assayed with anti-cit H3 antibodies. The antibody detected a specific band at 15 kDa, confirming the presence of cit H3 and MoETs (Figure 5A). cit H3 was not detected in human chromosomal DNA isolated by standard methods from THP-1 cells or from human blood monocytes. To test the antifungal activity of the isolated MoETs, the isolated MoETs were added to *C. albicans* and fungal growth was followed over 30 h by assaying the optical density at 604 nm. Treatment with this isolated extracellular trap DNA resulted in fungal growth inhibition. Fungal growth was reduced by about 45% after 6 h and by 35% after 24 h compared with *C. albicans* incubated in medium lacking DNA or incubated with monocyte or THP-1 chromosomal DNA (Figure 5A). Thus, MoETs isolated from human monocytes have antifungal activity and reduce *C. albicans* growth.

### MoET Formation Is Enhanced in the Presence of Active Human Serum

To determine the influence of serum factors on the extracellular trap formation, monocytes were incubated with *C. albicans* in 10% NHS or heat-inactivated 10% NHS, the released extracellular DNA was stained with SYTOX green, and the amount of released DNA was determined every hour for a total of 5 h. Monocytes cocultivated with *C. albicans* in the presence of NHS and inactive NHS released DNA into the medium, although presence of NHS aided the process. DNA release increased 5-fold after 1 h of incubation and 58-fold after 4 h of incubation compared with monocytes incubated in active serum but without *C. albicans* (Figure 5B). Neutrophils under the same conditions released 11-fold more DNA after 4 h in active NHS than neutrophils in active serum without *C. albicans*, but released 70–80% less DNA in total than monocytes. DNA release by neutrophils did not significantly differ in active and inactive NHS (Figure 5B). In summary, when challenged with *C. albicans*, monocytes released fourfold more DNA into the medium than neutrophils.

### Extracellular Traps from Monocytes and Neutrophils Activate the Complement System

**Figure 6:**
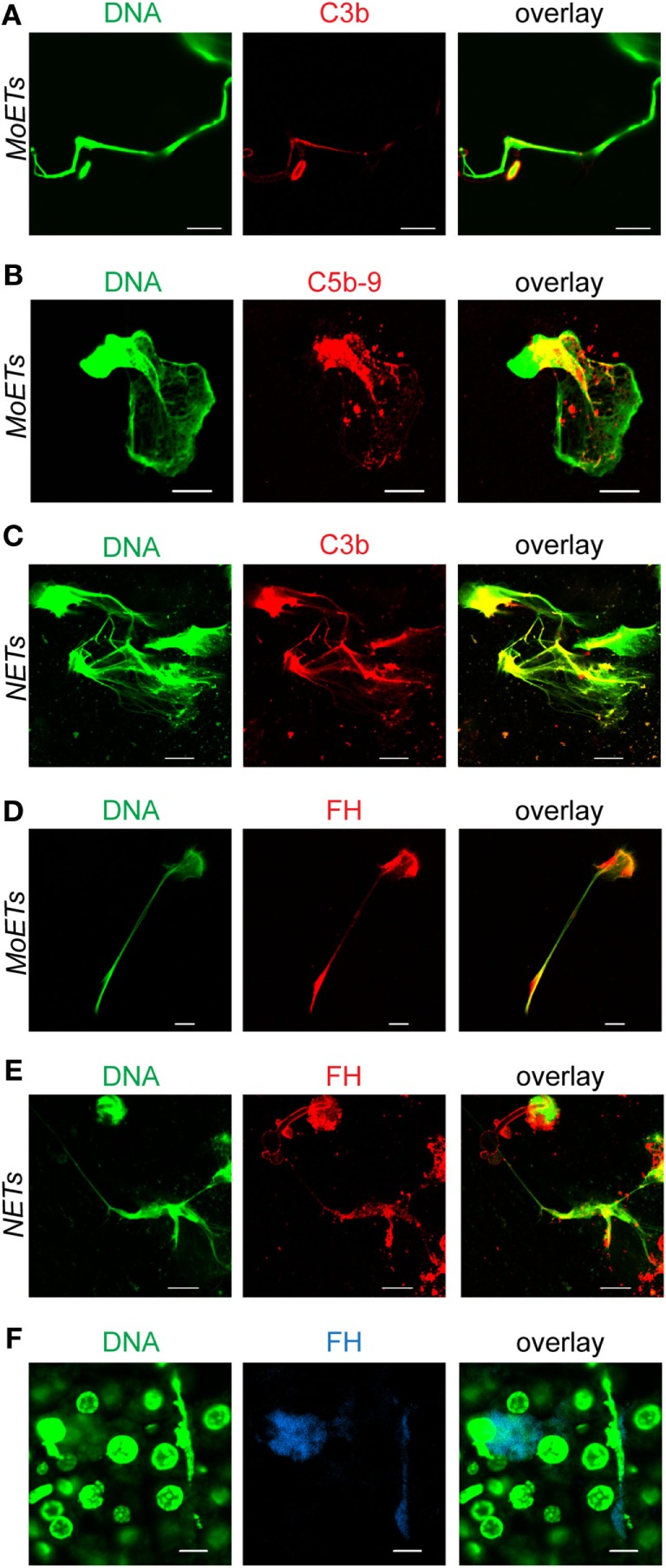
**Deposition of complement proteins on extracellular traps**. **(A)** Complement C3b and **(B)** complement C5b–9 are deposited on extracellular traps derived from monocytes (monocytic extracellular traps: MoETs) **(C)** or from neutrophils (neutrophil extracellular traps: NETs). Factor H (FH) binds to both **(D)** MoETs and **(E)** NETs. Human blood monocytes or neutrophils were incubated with *Candida albicans* (1:1) in 10% normal human serum for 4 h, fixed, and stained with the specific antibodies. Green: DNA, red: C3b, C5b–9, or FH. **(F)** Mouse FH binds to extracellular traps in mouse liver sections derived from *C. albicans*-infected mice. Sections were prepared 24 h after infection with *C. albicans*. Blue: FH; green: DNA. Pictures were taken with a laser scanning microscope LSM 710 (Zeiss) and processed using ZEN 2011 software (Zeiss). Scale bar: 10 µm. Experiments were repeated three times and representative images are shown.

To investigate whether MoETs activate complement in human serum, monocytes were incubated with *C. albicans* and active human serum. After incubation and immobilization on coverslips, the samples were stained with C3b and C5b–9 antibodies and investigated by laser scanning microscopy analysis. C3b and C5b–9 were deposited on MoETs as seen by colocalization of SYTOX green staining of the MoETS with C3b (Figure 6A) and C5b–9 (Figure 6B). Similarly, NETs showed C3b deposition (Figure 6C). To find out whether complement regulator FH recognizes and binds C3b on the DNA, the traps were immunostained with FH antibodies. Both extracellular traps from monocytes and neutrophils bound FH from the serum (Figures 6D,E) indicating restriction of complement activation by FH on the surfaces of the traps. Having shown that human extracellular DNA traps bind FH *in vitro*, we aimed at identifying DNA traps with FH in tissue of C. *albicans*-infected mice. Therefore, ultra-thin sections of embedded liver derived from *C. albicans*-infected mice (24 h post-infection) were stained to detect DNA traps with FH. Extracellular DNA was identified in the sections which also stained with FH antibodies (Figure 6F). These results confirmed that FH bound to released nuclear DNA in the liver tissue.

**Figure 7:**
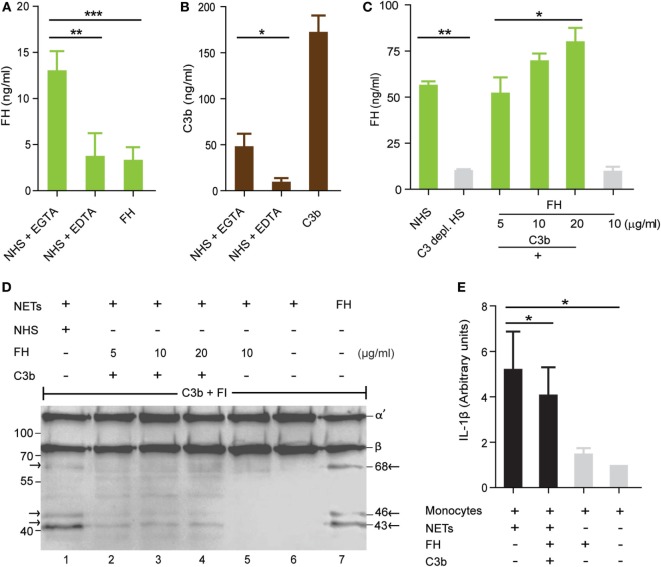
**Factor H (FH) bound to neutrophil extracellular traps (NETs) is functionally active and reduces inflammation induced by NETs**. **(A)** FH in normal human serum (NHS) binds to immobilized NETs. This binding is dependent on complement activation, as FH alone or in complement inactive serum (EDTA) does not bind. Immobilized NETs were incubated in 10% serum + EGTA, 10% serum + EDTA, or purified Factor H (10 µg/ml). FH, factor H. **(B)** C3b binds directly to the NETs. C3b (20 µg/ml) was incubated with the purified immobilized NETs and detected with anti C3. **(C)** Purified FH (5–20 µg/ml) binds together with C3b (20 µg/ml) to the NETs, but not alone. Factor H in NHS (10%) but not in C3-depleted NHS (10%) binds to the NETs. Data in (A–C) represent mean values ± SDs of three independent experiments (*p < 0.05, **p < 0.01, ***p < 0.001; two sided Student’s *t*-test). **(D)** FH bound to the NETs retains cofactor activity for factor I to cleave C3b. Western blot shows C3 cleavage products at 68, 46, and 43 kDa when NETs were incubated in 10% serum and also when factor H bound *via* C3b to the NETs and was incubated with C3b and factor I (FI) (lane 1–4). Incubation of NETs with FH alone before addition of C3b and FI shows no C3 cleavage bands (lane 5). NETs alone do not cleave C3b (lane 6). FH without NETs and incubated with C3b and FI results in cleavage of C3b (lane 7). Image is a representative result of three independent experiments. **(E)** Incubation of NETs with monocytes for 20 h induces interleukin-1 beta (IL-1β) secretion by isolated blood monocytes. FH bound by C3b on the NETs reduces the IL-1β secretion by monocytes. IL-1β level in the supernatant was measured by ELISA. Data represent mean values ± SDs of three independent experiments (*p < 0.05, one-way ANOVA).

To confirm *in vitro* binding of FH to the extracellular traps, NETs were isolated and immobilized to a microtiter plate *via* coated antibodies to citrullinated H3. The isolated immobilized NETs were incubated in NHS (10%) or with FH (10 µg/ml) and binding of FH was determined by ELISA. FH alone bound weakly to the NETs but attached to the NETs when recruited from NHS (Figure 7A). Complement inhibition by the addition of EDTA or EGTA to NHS substantially reduced FH binding to the NETs and suggested that FH was recruited by C3b to the NETs. Therefore, NETs were incubated in NHS activated *via* the alternative pathway and this time stained for C3b binding. C3b deposited on the NETs and binding was blocked by adding the complement inhibitor EDTA. Also, purified C3b bound to the NETs (Figure 7B). FH binding to the NETs from human serum was substantially reduced when NETs were incubated in C3-depleted serum compared to NHS (Figure 7C). FH alone did not bind to the NETs but together with C3b FH bound in a dose-dependent manner. Thus, FH binding to NETs is mediated by deposited C3b. To determine whether bound FH on the NETs is active, cofactor activity of bound FH was followed by Western blot analysis. Again NETs were immobilized to the microtiter surface *via* anti-cit H3 and incubated with C3b and FH. After washing off the unbound FH and C3b, NETs were incubated with C3b and factor I. C3 cleavage fragments in the supernatant were then detected by Western blot analysis. NETs bound FH-mediated cleavage of C3b by factor I as seen by C3b cleavage products at 68, 46, and 43 kDa (Figure 7D). Similarly, C3b was cleaved to iC3b, when NETs were incubated with NHS. These results demonstrate that NETs bound FH is functionally active and can mediate complement cofactor activity.

### FH Bound to NETs Inhibits a Pro-inflammatory Cytokine Response of Monocytes

Extracellular DNA traps often stay for a period of time in the blood before they are cleaved by DNase and are engulfed by phagocytes (28). Complement opsonization enhances the uptake of microbes and apoptotic cells by phagocytes (29). However, in contrast to microbes apoptotic self-cells do not induce the inflammasome and do not secrete inflammatory cytokines like IL-1β (30). Clearance of extracellular DNA traps by macrophages was previously shown not only to induce inflammatory cytokines (31) but also to act non-inflammatory (28). The presence of microbes, antimicrobial peptides, or microbial substances like LPS in the traps induces an inflammatory response in macrophages. Here, we isolated NETs from PMA induced neutrophils, incubated these NETs with human monocytes and IL-1β concentrations were determined after 20 h in the supernatants by ELISA. IL-1β production by PMA isolated with the NETs was excluded (Figure S5 in Supplementary Material). NETs induced the inflammasome in monocytes as determined by higher IL-1β concentrations in the supernatants compared to monocytes alone. Binding of FH *via* C3b on the NETs reduced IL-1β secretion in monocytes (Figure 7E). In contrast, FH alone added to the NHS did not modulate the IL-1β response in monocytes (data not shown). Altogether, the data demonstrate that binding of FH to NETs inhibits the inflammatory response of monocytes.

## Discussion

The current study demonstrates that human blood monocytes before differentiating into macrophages release extracellular DNA upon interaction with *C. albicans* to trap and attack the fungus. Upon contact with *C. albicans* for 2–4 h, monocytes decondense their nuclear DNA and release the DNA, which forms MoETs that cover and fix *C. albicans* cells and hyphae. Characterizing these traps in detail revealed many similarities to NETs because MoETs contain cit H3, elastase, MPO, and LAC (32), which altogether have antifungal activity to restrict *C. albicans* dissemination. Monocytes also form MoETs in response to other microbes (e.g., *E. coli* and *S. aureu*s); thus, trapping of microbes represents a direct immune reaction by monocytes. Extracellular traps activate complement and become opsonized with C3b. Complement regulator FH binds to opsonized traps and restricts complement activation and inflammation.

The current study aimed to shed light on how human monocytes react toward the pathogenic fungus *C. albicans*. Live time imaging revealed that monocytes phagocytosed *C. albicans* cells and this process was followed by monocytic DNA release. The released DNA formed fiber-like structures that enwrapped *C. albicans* cells and hyphae, as shown by laser scanning microscopy as well as electron microscopy. Caspase 1 inhibition in monocytes and subsequent incubation with *C. albicans* also resulted in MoETosis (data not shown) excluding DNA release by pyroptosis (33). Extracellular trap formation was first identified and described in neutrophils in response to microbes, and these traps are called NETs (34). During this process, chromatin decondensation leads to loss of the lobulated nucleus. Then, disintegration of intracellular membranes allows chromatin and extranuclear proteins to mix. The final step is the release of chromatin filaments decorated with proteins into the extracellular medium (25). The finding that human monocytes respond to *C. albicans* similar to neutrophils with the release of chromosomal DNA is new and supported by the observations that DNA release by monocytes was observed to *E. coli* (35) and to parasites *Besnoitia besnoiti* and *Eimeria boxis* (36). Besides neutrophils (24), eosinophils, mast cells, and macrophages also form extracellular traps (35, 37, 38). As shown here, *in vivo* trap formation thus represents a conserved immune reaction by immune cells toward microorganisms. However, MoET induction in monocytes is different to extracellular trap formation in macrophages, as DNA release in human monocytes cannot be triggered by cholesterol synthesis inhibitor simvastatin (data not shown) as previously reported for macrophages (39). Similar to METs (38) but in contrast to NETs, MoETs are not induced by LPS or PMA (latter not shown). Blocking the dectin receptor on monocytes by WGP also did not induce extracellular trap release. Thus, monocytes react specifically to microbes like *C. albicans* or *E. coli* by forming DNA traps.

The traps formed by monocytes contain cit H3. In neutrophils, citrullination of histone proteins is a crucial step in trap formation and is considered to be one of the markers of extracellular traps formed by neutrophils. Peptidylarginine deaminases catalyze histone citrullination, where the positively charged arginine residues are converted into polar uncharged citrulline side chains by deamination (40, 41). Similar peptidylarginine deaminase activities are expected to catalyze the citrullination reaction in monocytes.

Antimicrobial molecules such as MPO and elastase, which are components of azurophilic granules in monocytes, were found in MoETs. These components together with released LAC seem to be responsible for the killing of *C. albicans* by MoETs (34). MoETosis identified and described in the current study represents an active immune response of monocytes to *C. albicans*, which leads to monocytic cell death. The process of extracellular trap formation in monocytes differs significantly from apoptosis. MoETosis started with intracellular vacuole formation, followed by loss of the nuclei lobular structure. Chromatin decondensed in the nucleus, expanded, and filled the intracellular space before being released into the extracellular space. During this process, the cells remained alive, as observed by the absence of PS exposure on the plasma membrane (Figure S2 in Supplementary Material). Upon microbial contact, DNA was released within 2–4 h. Similarly, NETosis by neutrophils was characterized as a new form of cell death with clearly different characteristics to the pathway characterized for apoptosis (25).

In contrast to MoETosis, bleb formation marks the start of apoptosis in monocytes after 4–6 h. Soon after bleb formation, the membrane flip-flops the inner leaflet of the plasma membrane exposing PS. When apoptotic cells are not cleared by phagocytes, the apoptotic process can lead to secondary necrosis. Secondary necrosis is observed after 9–12 h and is accompanied by cell enlargement and disintegration of nuclear material. Thus, apoptosis shows clear distinguishable morphological features from MoETosis in monocytes. Altogether, MoETosis represents an early coordinated process upon exposure to *C. albicans* or other pathogens. By contrast, apoptosis starts with bleb formation after about 4.5 h, followed by secondary necrosis about 10 h later.

Monocytes released approximately fivefold more DNA in response to *C. albicans* than neutrophils under the same conditions. The higher amount of released DNA is to some degree explained by the counteraction of *C. albicans*. *C. albicans* follows several strategies to evade human immune attack (42–44). The most prominent evasion mechanism is the morphological switch to hyphal growth. With these hyphae, *C. albicans* can pierce through human cell membranes (45) and thus can also kill some monocytes during coincubation.

Altogether, the presented results highlight a prominent capacity of monocytes to form extracellular traps against *C. albicans* to inhibit growth and dissemination of the fungus. This trap formation is in agreement with the observation that the absence of monocytes in mice has detrimental effects on fungal infections (15) and that depletion of mononuclear phagocytes results in accelerated fungal proliferation in tissues and in increased mortality (46). Therefore, trap formation by monocytes makes a significant contribution in hindering from very early on the propagation of an infection. Indeed, in the case of infections, monocytes are recruited from the bone marrow and the number of monocytes increases in blood. In addition, monocytes are recruited to infection sites in tissues and inhibit *C. albicans* growth both *in vitro* and *in vivo* (47).

Once extracellular traps are formed, these structures activate complement. This is in agreement with recent reports of NETs (48, 49) and suggested that complement is regulated on the NETs to allow opsonization and phagocytosis to terminate the immune response. Clearance of extracellular traps is achieved with the help of DNase I, which cleaves the extracellular DNA and facilitates uptake and clearance of DNA by macrophages (28). Here, we show that extracellular traps from both monocytes and neutrophils activate the complement system (50) and that FH binds to the DNA traps *via* C3b. Bound to NETs FH retains physiological functions and exerts cofactor activity for the protease factor I to cleave C3b into iC3b. Furthermore, FH on the traps inhibits IL-1β secretion by monocytes. Thus, FH dampens the immune response to reduce the inflammatory response and to allow clearance of the traps.

## Author Contributions

CS designed and supervised the study. LH, MA, and EJ performed the experiments and discussed the data. MW performed EM analyses and read the manuscript. IJ performed the mouse infection experiments and provided tissue sections. NB generated a monoclonal mouse Pra1 antibody. All the participants discussed the data, and CS and PZ wrote the manuscript.

## Conflict of Interest Statement

The authors declare no financial or commercial conflicts of interest.

## Abbreviations

MoET, monocytic extracellular trap; E, elastase; MPO, myeloperoxidase; LAC, lactoferrin; cit H3, citrullinated histone 3; NET, neutrophil extracellular trap; FH, factor H; IL-1β, interleukin-1 beta.
