# Supplementary material for: Factor H Binds to Extracellular DNA Traps Released from Human Blood Monocytes in Response to Candida albicans
Source: Front Immunol. 2017 Jan 13;7:671. doi: 10.3389/fimmu.2016.00671 (PMC5233719; doi:10.3389/fimmu.2016.00671)
Supplement: Supplementary file 2 [file data_sheet_1.pdf]

Supplementary Material

Factor H binds to extracellular DNA traps released from human blood monocytes in response to *Candida albicans*

Luke D. Halder[1], Mahmoud A. Abdelfatah[1], Emeraldo A.H. Jo[1], Ilse D. Jacobsen[2,3], Martin Westermann[4], Niklas Beyersdorf[5], Stefan Lorkowski[6], Peter F. Zipfel[1,3] and Christine Skerka[1]

*** Correspondence:** Christine Skerka: Christine.skerka@hki-jena.de

Supplementary Data

Supplementary Figure 1. Human neutrophils release extracellular DNA (NET) to trap *C. albicans.*

Supplementary Figure 2. Human monocytes form extracellular DNA traps in response to hyphae locked *C. albicans (cph1/efg1)* cells.

Supplementary Figure 3: Physiological changes during MoETosis versus apoptosis in monocytes

Supplementary Figure 4. Human monocytes express CD14 and human neutrophils CD66b

Supplementary Figure 5. NETs cause the release of IL-1β from human monocytes.

1.1 Supplementary Figures

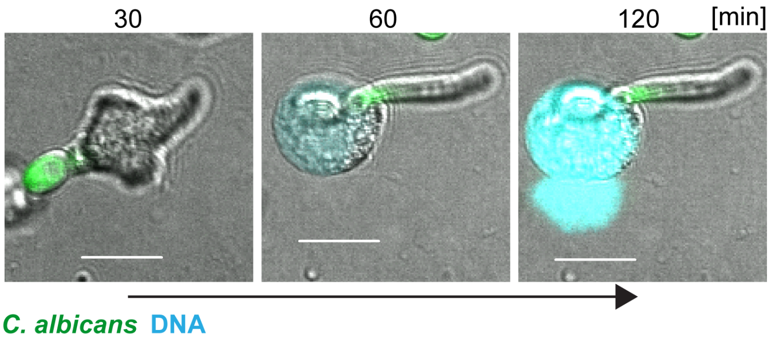

Supplementary Figure 1. Human neutrophils release extracellular DNA (NET) to trap *C. albicans*.

Human neutrophils were incubated with GFP-*C. albicans* (1:1) in 10 % NHS and DNA release was followed over 2 h by live cell imaging. Released DNA was stained with SYTOX blue. Pictures were taken with a LSM710 microscope (Zeiss), fitted with a 40x, 1.4 NA, oil-immersion lens, and processed, using ZEN 2011 software (Zeiss). Scale bar: 10 µm .

Method

Neutrophils were isolated from fresh human blood from healthy male donors using Ficoll-Paque PLUS density gradient centrifugation. Erythrocytes were removed by 3% dextran precipitation followed by hypotonic lysis with sterile water. Isolated neutrophils were suspended in growth medium and used within 1 h. Purity (>95 %) was determined by flow cytometry using Alexa fluor 488 anti-human CD16 antibody (Biolegend, London, UK) and Alexa fluor 647 anti-human CD66b antibody (Biolegend, London, UK). For live cell imaging neutrophils (2×10^5) were seeded on Poly-L-lysine coated 30 mm culture dishes and incubated in growth medium with 10% NHS at 37°C with

5% CO_2. GFP-expressing *C. albicans* (2×10^5) were added to the seeded cells. DNA release was detected with nucleic acid dye SYTOX blue (ThermoFisher Scientific, Dreieich, Germany) (5 µM) during co-incubation. Neutrophils were co-incubated in small culture dishes at 37°C with 5% CO_2 and subjected to confocal laser scanning microscopy ((CLSM) – LSM 710 from Carl Zeiss, Jena, Germany). Live time images were captured over 6 h taking images every 30 sec using ZEN 2011 (magnification 40x, 444⁄480 nm for SYTOX blue, 488/509 nm for GFP). The experiment was repeated 2 times.

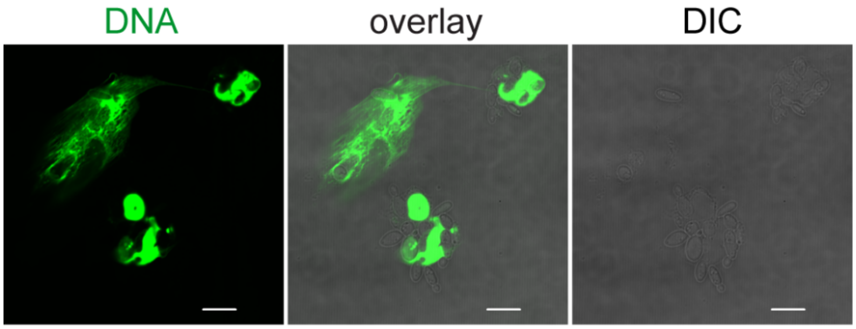

Supplementary Figure 2.

Human monocytes form extracellular DNA traps in response to hyphae locked *C. albicans* (*cph1/efg1*) cells. Human monocytes were incubated with hyphae locked *C. albicans* (1:1) cells for 4 h and stained for extracellular DNA traps with SYTOX green. Pictures were taken with a LSM710 microscope (Zeiss), fitted with a 63x, 1.4 NA, oil-immersion lens, and processed, using ZEN 2011 software (Zeiss). Scale bar: 10 µm.

Fixed cell imaging. *C. albicans* cph1Δ/efg1Δ cells were kindly provided by B. Hube (HKI Jena, Jena, Germany). *C. albicans* cph1Δ/efg1Δ cells were added to monocytes (5×10^5) on 13 mm Poly-L-lysine coated coverslips in a 24 well plate and incubated for 4 h in growth medium with 10% NHS at 37°C in 5% CO_2. The medium was aspirated and reactions were fixed using 4% paraformaldehyde (PFA) for 10 min. After treatment with PFA, coverslips were stained with 5 µM SYTOX green for detection of MoETs. Images were captured using LSM 710 equipped with ZEN 2011 (504/523 nm for SYTOX green). The experiment was repeated 2 times.

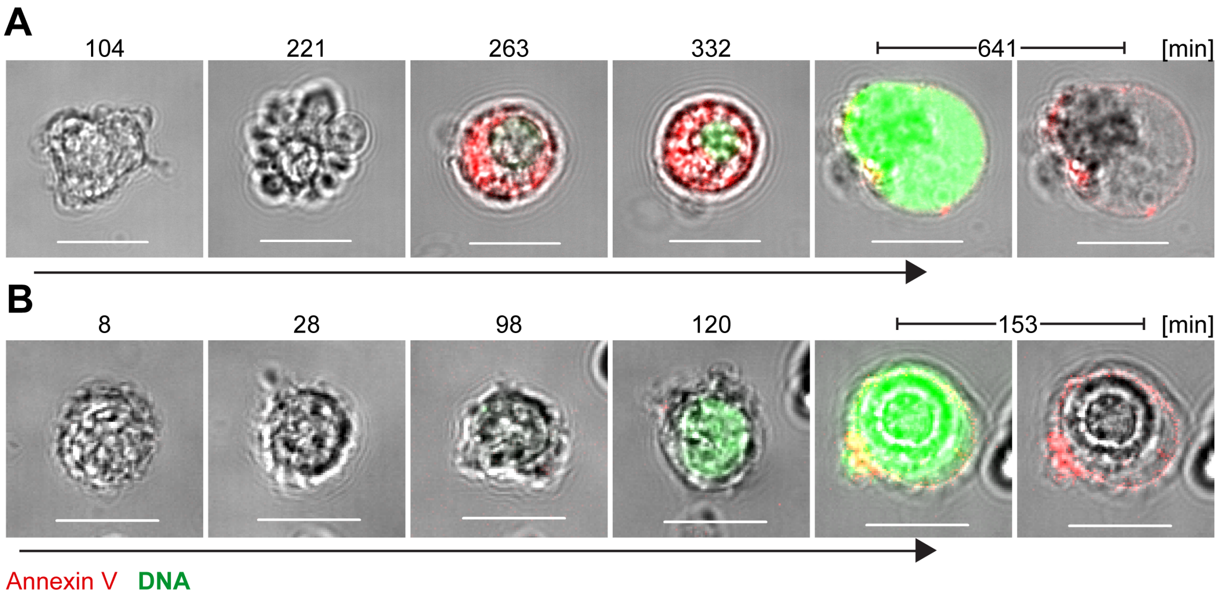

Supplementary Figure 3: Physiological changes during MoETosis versus apoptosis in monocytes (A) Apoptotic monocytes show bleb formation after 220 min and stain for PS exposure (red). DNA release (green) from apoptotic cells (secondary necrosis) occurs after 640 min. **(B)** During MoET formation vacuoles are formed after about 30 min upon interaction with *C. albicans*, followed by decondensation of DNA (green) and release of the DNA after 150 min. PS staining (red) occurs after DNA release. Pictures were taken with LSM710 (Zeiss), 40x, 1.4 NA, oil-immersion lens, using ZEN2011 software (Zeiss). Scale bar: 10 µm.

For live cell imaging of MoETosis, monocytes (2×10^5) were seeded on Poly-L-lysine coated 30 mm culture dishes and incubated with *C. albicans* wild type (SC5314) (2×10^5) in growth medium with 10% NHS at 37°C with 5% CO_2. Cell death of monocytes was detected with Alexa Fluor 647 labeled Annexin V (1:100) (Biolegend, London, UK) and 5 µM SYTOX green. To follow apoptosis of monocytes cells were incubated in plain medium in small culture dishes at 37°C with 5% CO_2 and subjected to confocal laser scanning microscopy. Live time images were captured every 30 sec over

20 h using ZEN 2011 (magnification 40x, 504/523 nm for SYTOX green and 594/633 nm for Alexa Fluor 647). The experiment was repeated 3 times.

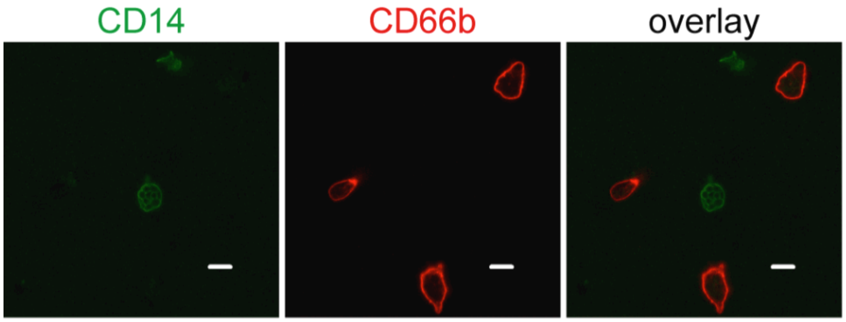

Supplementary Figure 4. Human monocytes express CD14 and human neutrophils CD66b. Human monocytes and human neutrophils were co-incubated and stained for CD14 and CD66b. CD14 (green) but not CD66b was detected on monocytes. CD66b (red) but not CD14 was detected on neutrophils. Pictures were taken with LSM710 (Zeiss), 40x, 1.4 NA, oil-immersion lens, using ZEN2011 software (Zeiss). Scale bar: 10 µm.

For live cell imaging neutrophils (1×10^5) and monocytes (1×10^5) were seeded on Poly-L-lysine coated 30 mm culture dishes and incubated in growth medium with 10% NHS at 37°C with 5% CO_2. Cells were stained for CD14 and CD66b using Alexa Fluor 488 anti-human CD14 (1:100) and Alexa Fluor 647 anti-human CD66b antibodies (1:100) (Biolegend, London, UK). Live time images were captured after 30 min of incubation using ZEN 2011 (magnification 40x, 444/480 nm for SYTOX blue, 488/509 nm for GFP). The experiment was repeated 2 times.

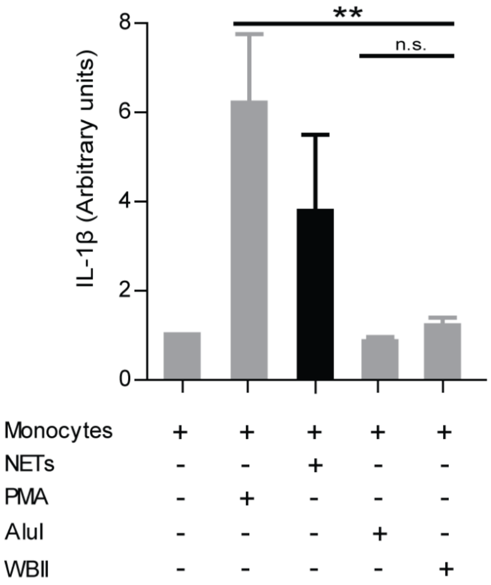

Supplementary Figure 5. NETs cause the release of IL-1β from human monocytes. Incubation of isolated human blood monocytes with isolated NETs for 20 h induces IL-1β secretion. NETosis was induced in human neutrophils with PMA. PMA induces IL-1β secretion in monocytes. Washing of isolated NETs (WBII: second wash buffer with DPBS) as well as AluI (enzyme to digest NETs into fragments) do not induce IL-1β release by monocytes. (**$p < 0.01$, n.s.-non-significant; Student's two-tailed t-test and one way ANOVA.)

For isolation of extracellular traps neutrophils (1.5×10^6) were seeded into 12 well plates and incubated in growth medium with 100 nM phorbol 12-myristate 13-acetate (PMA) (Sigma-Aldrich, Taufkirchen, Germany) for 4 h at 37^0C and 5% CO_2. Neutrophils alone were used as controls. Following 4 h incubation the wells were washed twice with DPBS and extracellular traps were digested into fragments by adding AluI (4 U/ml) (ThermoFisher Scientific, Dreieich, Germany) for 20 min at 37°C. The supernatants were harvested after centrifugation (10,000 x g for 5 min at 4°C). Blood derived monocytes were incubated alone, with 100 nM of PMA or in presence of second wash

(WBII), or with AluI (2 U/ml) or isolated NETs in growth medium for 20h and IL-1β levels were assayed with ready-set-go ELISA kit (eBioscience, Frankfurt, Germany) according to manufacturer's protocol. The IL-1β level measured with monocytes alone was set as 1. The experiment was repeated 3 times with three different blood donors.
